# Development of Light-Responsive Poly(γ-Benzyl-L-Glutamate) as Photo Switches by a One-Step NCA Method

**DOI:** 10.3389/fchem.2020.00591

**Published:** 2020-08-04

**Authors:** Pin Chen, Jingyang Kong, Xin Wang, Weiye Ma, Xia Yang, Yuqing Qin, Xiaohong Hu

**Affiliations:** School of Material Engineering, Jinling Institute of Technology, Nanjing, China

**Keywords:** PBLG, NCA method, photo switch, light response, azobenzene

## Abstract

Synthesized polypeptide is attracting an increased interests due to its excellent biological characteristic and adjustable chemical properties in bio-related fields. But polypeptide itself has no switching properties, which is harmful to the development of its application as a control component. Herein, light-responsive poly(γ-benzyl-L-glutamate)s (PBLGs) is synthesized by a one-step NCA method using p-aminoazobenzene (m-AZO) and p-diaminoazobenzene (m-DAZO) as initiators. PBLGs exhibit amorphous characteristics with obvious T_g_ transition, which are 14°C for PBLG1 and 21°C for PBLG2. In order to forecast the structure-property information of PBLGs, theoretical UV-vis spectra as well as the energy gap between HOMO and LUMO is calculated by DFT calculation. Experimental results of UV-vis spectra exhibit similar characteristics to those of theorical UV-vis spectra except for the 40–50 nm red-shifting of absorbance peak. Furthermore, the absorbance intensities of PBLGs have a good linear relationship with their concentration, but their linearity range depending on concentration is completely different. Then, trans–cis transition under a different excitation source and cis–trans recovery in a dark environment are tracked in real-time by UV-vis spectra to evaluate the light response performances. It is found that UV light is the only effective excitation source for PBLG1, and blue light is another effective excitation source for PBLG2 besides UV light. Furthermore, the addition of alcohol and water as cosolvents has little effect on trans→cis transition in UV-light-excited systems, but it shortens recovery time of the cis→trans process in a dark environment. By contrast, the detectable isomerization process becomes unclear with the addition of alcohol in blue-light-excited system. Furthermore, either alcohol or water in solvents accelerate both the trans→cis and cis→trans process in a blue-light-excited system.

## Introduction

Polypeptide has a similar chemical component and secondary structure to a natural protein, which plays an essential role in biological activities (Li et al., 2017; Skoulas et al., 2017; Zhou et al., 2017; Editors, 2019; Duan and Li, 2020; Duong et al., 2020). This characteristic endows polypeptide with properties of low toxicity, good biodegradability, and good biocompatibility (Li et al., 2017; Skoulas et al., 2017; Zhou et al., 2017; Editors, 2019; Duan and Li, 2020; Duong et al., 2020). On account of these advantages, this kind of material brings an increasing amount of attention to bio-related fields like drug delivery, biosensors, biological diagnosis, and tissue engineering. Among polypeptides, synthesized polypeptides are the main focus due to their flexible and controllable molecular structure and are usually realized by biological synthesis, solid phase peptide synthesis (SPPS), and ring-open polymerization (ROP) based on the α-amino acid *N*-carboxyanhydrides (NCA method) (Wibowo et al., 2014; Shen et al., 2015; Zhou and Li, 2018). From the aspect of commercial process, the NCA method is more suitable for polypeptide materials due to the raw materials available, low cost, high yields, and short production cycle. More recently, Zn(OAc)_2_-catalyzing ROP of NCA was used to obtain well-defined polypeptides with controlled molecular weight (M_w_) and narrow molecular weight distribution (MWD) by Nie et al. (2018). Although the method has existed for more than 100 years, it is still a research hotspot for polypeptide synthesis (Wibowo et al., 2014).

Besides fundamental biological properties, synthesized polypeptide can be endowed with other properties like stimuli-responsive properties through post-synthesis functionalization in order to suit the needs of different applications (Dong and Chen, 2011; Shen et al., 2015; Qu et al., 2018; Xiao et al., 2019). Since efficiency and effectiveness were objects of chemical synthesis, tedious steps should be avoided for functional polypeptide preparation. Thus, a facile method to fabricate functional polypeptide with fewer steps is also needed to extend the application of polypeptides. With the development of the NCA method, the initiator of primary amine is considered to be a kind of idea initiator (Wibowo et al., 2014). Once an initiator is polymerized in the ROP process, it becomes a terminal group or medial unit of synthesized polypeptides depending on the number of the initiator's amino groups. Therefore, measures that combine functional groups with initiators are presumed to be a facile way to fabricate functional polypeptide based on the NCA method.

In recent decades, as a kind of stimuli-response material, photo switches have become new favorites in fields that require switching effects, including information science and chemical sensing due to structural changes in light. As a member of the photo switches, azobenzene (AZO) possesses two different reversible isomers (trans and cis) (Beharry and Woolley, 2011; Bian et al., 2016; Michael Kathan, 2017; Chen et al., 2018; Lerch et al., 2018; Pang et al., 2018, 2019). The reversible isomerization could be realized upon light excitation and thermal transition. But the thermal transition is influenced by either the external environment or internal structure of the AZO family, which bring problems of photobleaching, instability, and uncontrollability (Pang et al., 2018, 2019). In order to overcome problems, measures of substitute modifying, grafting, copolymerization, and immobilization have been used to improve the stability and controllability including our previous studies (Deka et al., 2015; Liu et al., 2015; Lin et al., 2016, 2017). Based on previous research, the long chains of polymers would help to increase the stability and controllability of thermal transition (Pang et al., 2018, 2019). Therefore, designing an AZO-functionalized polypeptide would provide a kind of practical photo switch for its application in bio-related fields.

In consideration of requests for an NCA synthesis method and photo switches, amino-substituted AZOs have been chosen as initiators for the ROP of NCA. Generally, γ-benzyl-L-glutamate (BLG) is a kind of readily available and low-cost amino acid with good performance (Cauchois et al., 2013; de Miguel et al., 2014; Sun et al., 2015). Also, it can be polymerized into poly(γ-benzyl-L-glutamate) (PBLG) by the NCA method through two steps, namely, synthesis of γ-benzyl-L-glutamate-*N*-carboxyanhydride (BLG-NCA) and ROP of NCA. In view of these facts, BLG was used for the design and synthesis of polypeptide photo switches in this study. Simultaneously, considering the steric hindrance in polymerization, para amino substituted AZOs were used in the work. In order to evaluate the performance of synthesized PBLG as photo switches, properties of reversible light responses and thermal recovery were investigated and analyzed in the research.

Although azobenzene-based photo switches have been designed and confirmed, the research provides an efficient and effective approach with which to synthesize an adjustable polypeptide photo switch. Moreover, the research provides basic data for clarification of isomerization mechanism and amendment of theoretical model for AZO-based photo switches.

## Experiment

### Material

γ-benzyl-L-glutamate (BLG), triphosgene, p-aminoazobenzene (m-AZO), and p-diaminoazobenzene (m-DAZO) were purchased from Aladdin. Tetrahydrofuran (THF), n-hexane, diethyl ether, ethyl acetate (EA), dimethylformamide (DMF), dioxane, and alcohol were obtained from Sinopharm Chemical Reagent Co., Ltd, China. All other reagents and solvents were of analytical grade and used as received.

### Synthesis and Characterization of BLG-NCA

γ-benzyl-L-glutamate-*N*-carboxyanhydride (BLG-NCA) was synthesized by a reaction between BLG and triphosgene. Briefly, 14 g BLG was dissolved in 200 mL anhydrous THF in reaction vessel with condensing reflux unit at stirring state. Then the solution was heated to 50°C, and 20 g triphosgene was added. The reaction continued until the solution turned from cloudy to clear. The reaction was cooled to room temperature, and N_2_ was concurrently inlet into the reaction system until the liquid volume did not decrease any more. The solution was precipitated by n-hexane. After being purified by EA/n-hexane and dried in a vacuum oven, BLG-NCA was obtained and characterized by ^1^H nuclear magnetic resonance (^1^H NMR, Bruker, AV300) using CDCl_3_ as a solvent.

### Synthesis and Characterization of PBLGs

PBLG was synthesized by ROP. Briefly, 1 g dried BLG-NCA was dissolved in 20 mL dioxane in an anaerobic environment, and 76 μmol initiator (m-AZO or m-DAZO) in DMF solution was added batches of eight by way of injection. After the reaction had lasted for 72 h, the reaction solution was precipitated by diethyl ether. After being purified by THF/diethyl ether and dried in a vacuum oven, PBLG was obtained. When the initiator was m-AZO, final PBLG was denoted as PBLG1. When the initiator was m-DAZO, final PBLG was denoted as PBLG2.

Synthesized PBLGs were characterized by ^1^H NMR (Bruker, AV300) using D6-DMSO as a solvent and differential scanning calorimetry detection (PerkinElmer, DSC 4000). Furthermore, the degree of polymerization (DP), namely the number of repeated units, was calculated according to relative normalized H content between structural unit and initiator residue, which was obtained from integration area of ^1^H NMR. Moreover, UV-vis spectra of PBLGs solution were tracked as a function of polymer concentration in order to characterize their characteristic peak and concentration dependence.

### Investigation of Effective Excitation Sources for PBLGs

PBLG (PBLG1 or PBLG2) was dissolved in DMF to obtain dilute solution. Four different excitation sources, which were a UV lamp (240-365 nm, 3.6 W), blue light lamp (450-457 nm, 3.6 W), yellow light lamp (580–595 nm, 3.6 W), and a red light lamp (620–625 nm, 3.6 W), were used to excite isomerization for two polymers with a light density of 1000 Lux and at room temperature, respectively. After light irradiation, the recovery process was realized in a dark environment at room temperature. Real-time UV-vis spectra were recorded the during irradiation process and recovery process, respectively. In the next step, repeated irradiation and recovery methods were applied to demonstrate fatigue resistance of PBLGs.

### DFT Calculations for Azobenzene-Based Molecules

The calculating model was constructed using an azobenzene-based structural unit (initiator unit) including nearby BLG for PBLG. All calculations were carried out with Gaussian 09 programs at a CAM-B3LYP/6-31G (d, p) level (Ransil, 1961; Boys and Bernardi, 1970; Frisch et al., 2009). The UV-vis spectra and the vertical excitation potential for the first singlet excited state (S1) were calculated by time-dependent DFT (TDDFT) calculations at the optimized S0 geometry at the same level. Solvent effects were taken into account within the polarizing continuum model (PCM) framework in all the geometry optimization and excited state calculations.

### Response-Recovery Performance of PBLGs

Besides excitation sources, the effects of solvents on the response-recovery performance of synthesized PBLGs were investigated. Besides DMF, water and alcohol were used to adjust the property of solvents. Similarly, the real-time UV-vis spectrum in the irradiation process as well as that in recovery time under a dark environment was recorded. Light density was set at 1000 Lux, and temperature was set at room temperature.

## Results and Discussions

### Synthesis of PBLGs

**Figure 1:**
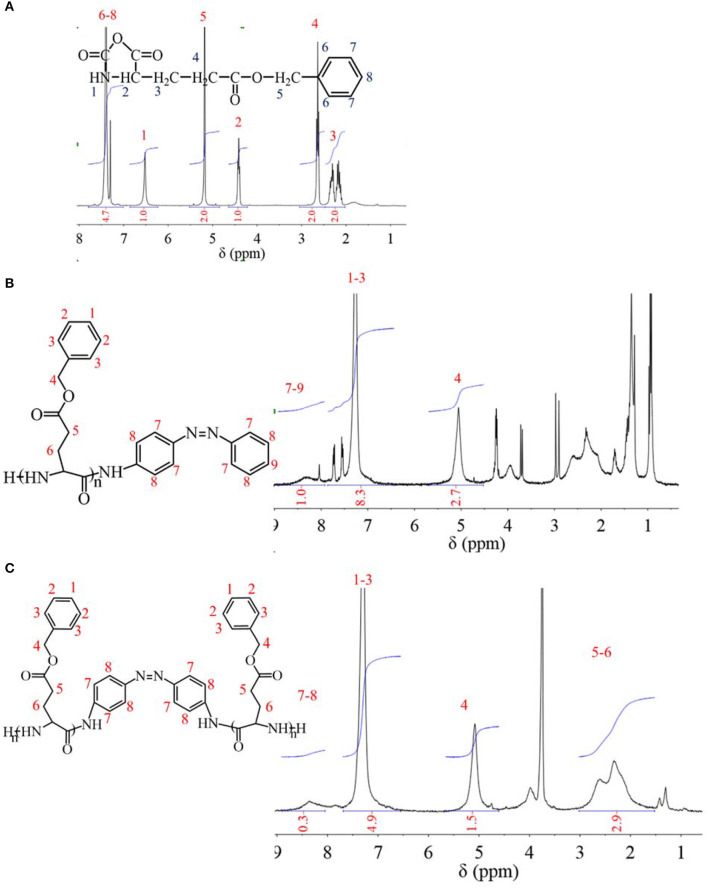
^1^H NMR spectra of **(A)** BLG-NCA monomer; **(B)** PBLG1; **(C)** PBLG2.

Before synthesis of PBLGs, a BLG monomer was synthesized by elimination reaction with participation of triphosgene. The ^1^H NMR spectrum of BLG is shown in Figure 1A, which was analyzed as follows: chemical shifts from 7.2 to 7.4 ppm were attributed to protons on benzene ring at positions 6–8 with an integration of 4.7; the chemical shift at 6.5 ppm was attributed to proton of secondary amine at position 1 with an integration of 1.0; the chemical shift at 5.2 ppm was attributed to protons of methylene at position 5 with an integration of 2.0; the chemical shift at 4.4 ppm was attributed to the proton of methyne at position 2 with an integration of 1.0; the chemical shift at 2.7 ppm was attributed to protons of methylene at position 4 with an integration of 2.0; the chemical shifts from 2.1 to 2.4 ppm were attributed to protons of methylene at position 3 with an integration of 2.0. Since areas of resonance peaks are proportional to the number of protons, the ratio of different peak area is equal to ratio of proton number at different positions. Therefore, both the existence of the -NH- group at position 1 and the H atom ratio of position 1 to other positions confirmed the successful synthesis of BLG-NCA. Furthermore, no other chemicals were found in the ^1^HNMR spectrum, which indicated high purity of synthesized BLG-NCA. Moreover, it was found from the experiment that the yield of this reaction for BLG-NCA was 70–75% w/w.

PBLG was obtained by the ROP method. When the initiator was m-AZO, AZO existed as a terminal group of PBLG1 (Figure 1B). The ^1^HNMR spectrum of PBLG1 is also shown in Figure 1B, which was analyzed as follows: the chemical shifts from 7.9 to 8.6 ppm were attributed to protons at positions 7–9, chemical shifts from 7.0 to 7.8 ppm were attributed to protons at positions 1–3; and a chemical shift at 5.1 ppm was attributed to protons of methylene at position 4. These results not only confirmed the successful synthesis of PBLG1, but it also provided quantitative information of DP, which was calculated to be 14 according to the average normalized peak area (integration, Figure 1B) ratio of BLG units (positions 1–4) to AZO residue (positions 7–9). When the initiator was m-DAZO, AZO existed as a medium group of PBLG2 (Figure 1C). Chemical shifts of the ^1^HNMR spectrum for PBLG2 in Figure 1C were attributed as follows: chemical shifts from 8.1 to 8.7 ppm belonging to protons at positions 7–8; chemical shifts from 6.6 to 7.6 ppm belonging to protons at positions 1–3; chemical shift at 5.1 ppm belonging to protons at position four; and chemical shifts from 1.6 to 3.0 ppm belonging to protons at positions −6. Also, the integration of these peaks was listed in Figure 1C. Similarly, the DP of synthesis PBLG2 was calculated to be 22.

### Characterization and Calculation of PBLGs

**Figure 2:**
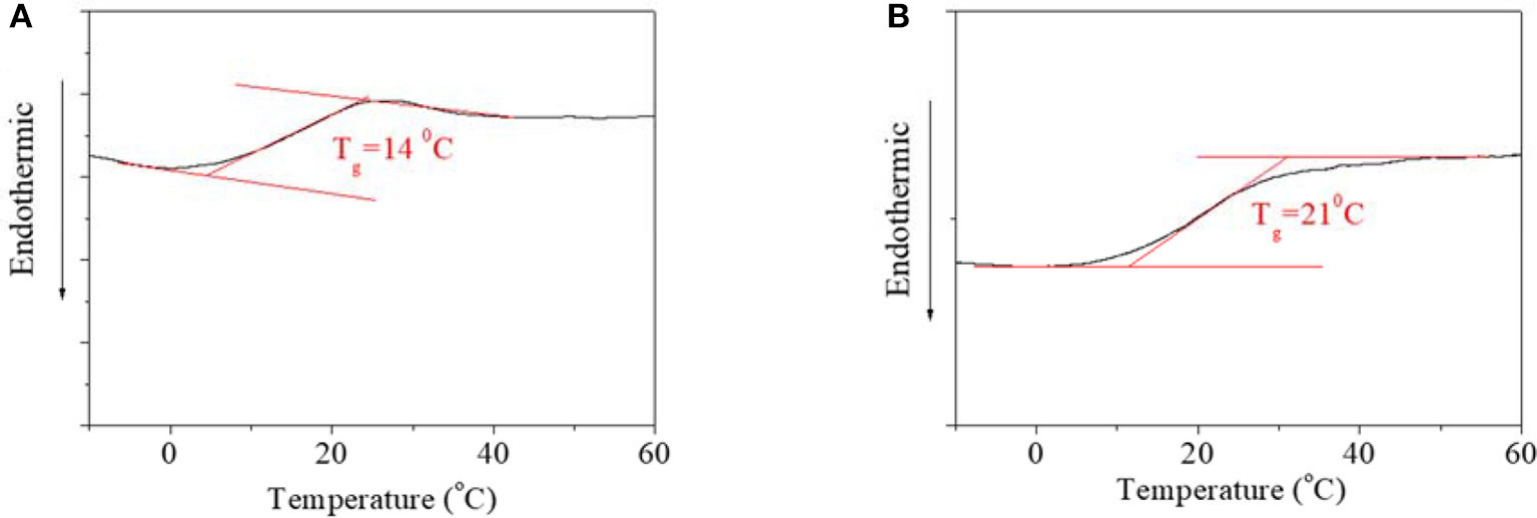
DSC curve of **(A)** PBLG1; **(B)** PBLG2.

DSC curves of PBLGs were detected to clarify structural characteristic, which are shown in Figure 2. No obvious phase transformation was found in the DSC curves of either PBLG1 (Figure 2A) or PBLG2 (Figure 2B), which indicated that the two polymers were both amorphous polymers. For PBLG1, an obvious T_g_ transition was found at 14°C (Figure 2A). For PBLG2, a T_g_ transition was found at 21°C (Figure 2B). Using common sense, we can see that the T_g_ transition was directly dependent on the length, free volume, and polarity of the domain. On account of the structural similarity of the two polymers, their polarities were also similar. Therefore, higher T_g_ might be the result of one of two things. One was that the domain length for PBLG2 was greater than that for PBLG1 if the DP of the polymers was not greater than one domain. The other was that the free volume of PBLG2 was smaller than that of PBLG1 in the same environment, which meant PBLG2 possessed a more compacted structure than PBLG1 due to the position of the AZO unit.

**Figure 3:**
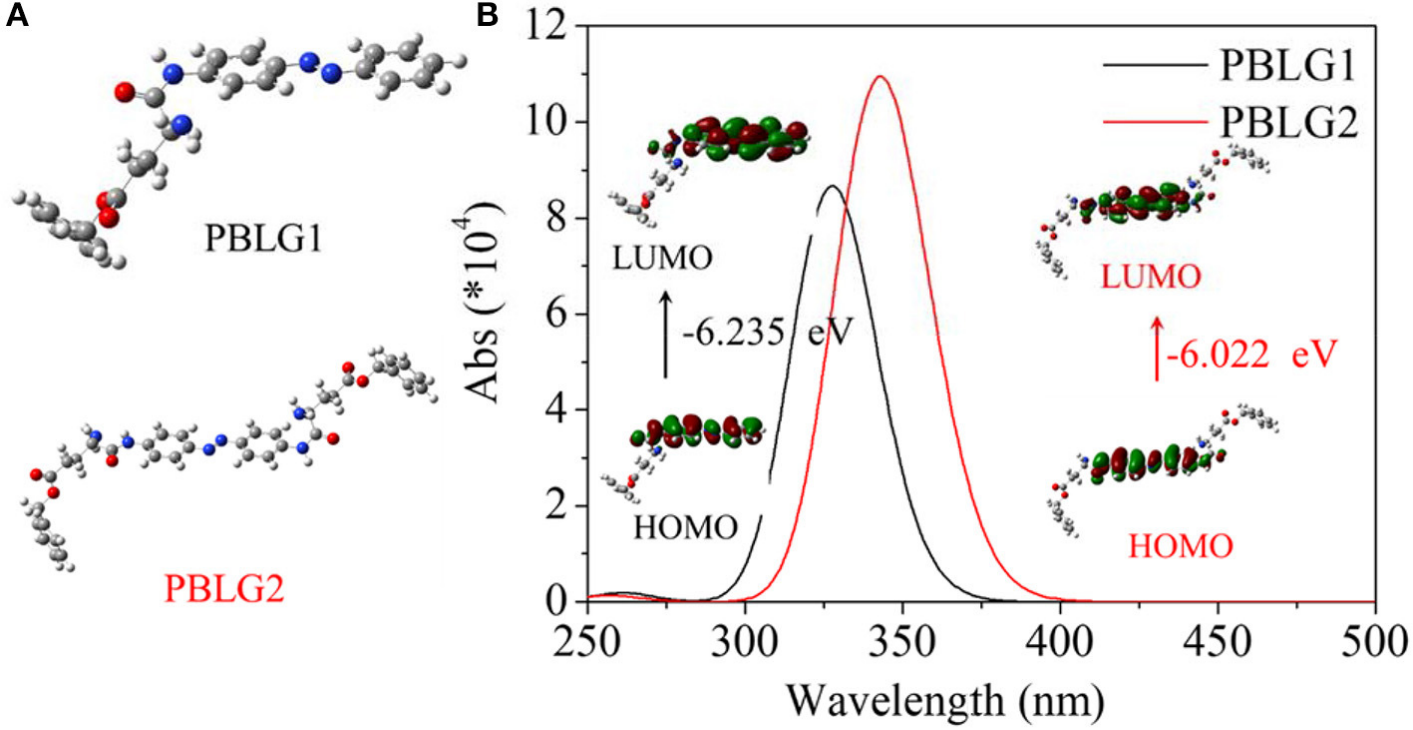
**(A)** Calculated models for PBLG1 and PBLG2; **(B)** Calculated UV-vis spectra and the corresponding molecular orbital transitions from HOMO to LUMO of two different models. The energy gap is illustrated in the figure.

In order to forecast isomerization characteristics for PBLGs, DFT was used to calculate UV-vis spectra and the corresponding molecular orbital transitions from HOMO to LUMO using simplified molecular models constructed by a central AZO unit and one or two nearby structural unit(s) (Figure 3A). Calculated results are shown in Figure 3B. It was found that PBLG1 had a maximum absorbance peak at 327 nm in theoretical UV-vis spectra, and PBLG2 had a maximum absorbance peak at 343 nm red-shifting 16 nm away from that of PBLG1. Besides the different of wavelength between two PBLGs, the absorbance intensity of PBLG2 was higher than that of PBLG1 of the same status. Furthermore, the energy gap between HOMO and LUMO for PBLG2 was also lower than that for PBLG1 (Figure 3B). According to existing theories, the maximum peak belonged to π-π^*^ transition for the trans-isomer of an AZO unit, and trans→cis transition included two process, from S0 up to S1 and from S1 down to S2 (base state for the cis isomer). Therefore, the excited energy of PBLG2 from S0 (base state for trans isomer) to S1 (excited state) was lower than that of PBLG1, which resulted in the absorbance peak red-shifting and absorbance intensity increasing since UV-vis absorbance is generally associated with electron transition. Simultaneously, the effective excitation wavelength for trans→cis transition was inferred to be related to maximum UV-vis absorbance wavelength.

**Figure 4:**
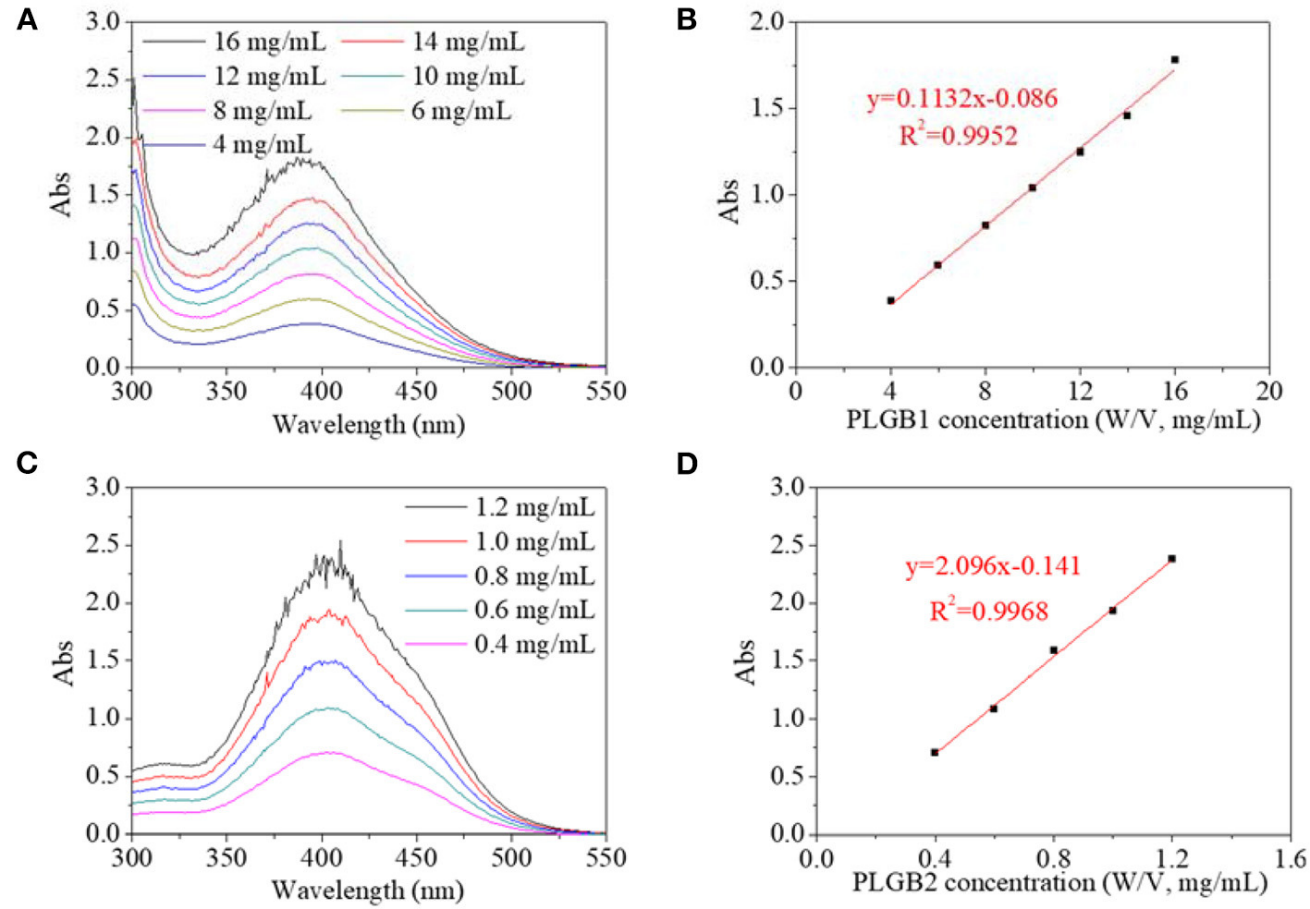
UV spectra of **(A)** PBLG1 and **(B)** PBLG2 with different polymer concentration; **(C)** absorbance of PBLG1 at 390 nm as a function of PBLG1 concentration; **(D)** absorbance of PBLG2 at 400 nm as a function of PBLG2 concentration.

In an experimental system, UV spectra of PBLGs in DMF solution and their concentration dependence were characterized and shown in Figure 4. On the UV spectrum, PBLG1 exhibited a maximum absorbance peak at 390 nm, and the absorbance increased with PBLG1 concentration (Figure 4A). Similarly, the maximum absorbance peak for PBLG2 occurred at 400 nm and also increased with its concentration (Figure 4C). The experimental position of the maximum absorbance peak was 55–70 nm, red-shifted from a theoretical position, which was reasonable, as that theoretical calculation could not involve all effects of a practical experiment. But the tendency to red-shift would not change. Furthermore, the absorbance intensity exhibited a good linear relationship with the polymer concentration (Figures 4B,D). The absorbance intensity of PBLG2 was, however, several times that of PBLG1 in the same concentration. Additionally, the linearity would help to quantify the polymer in terms of its application as a standard method.

### Effective Excitation Sources for PBLGs

**Figure 5:**
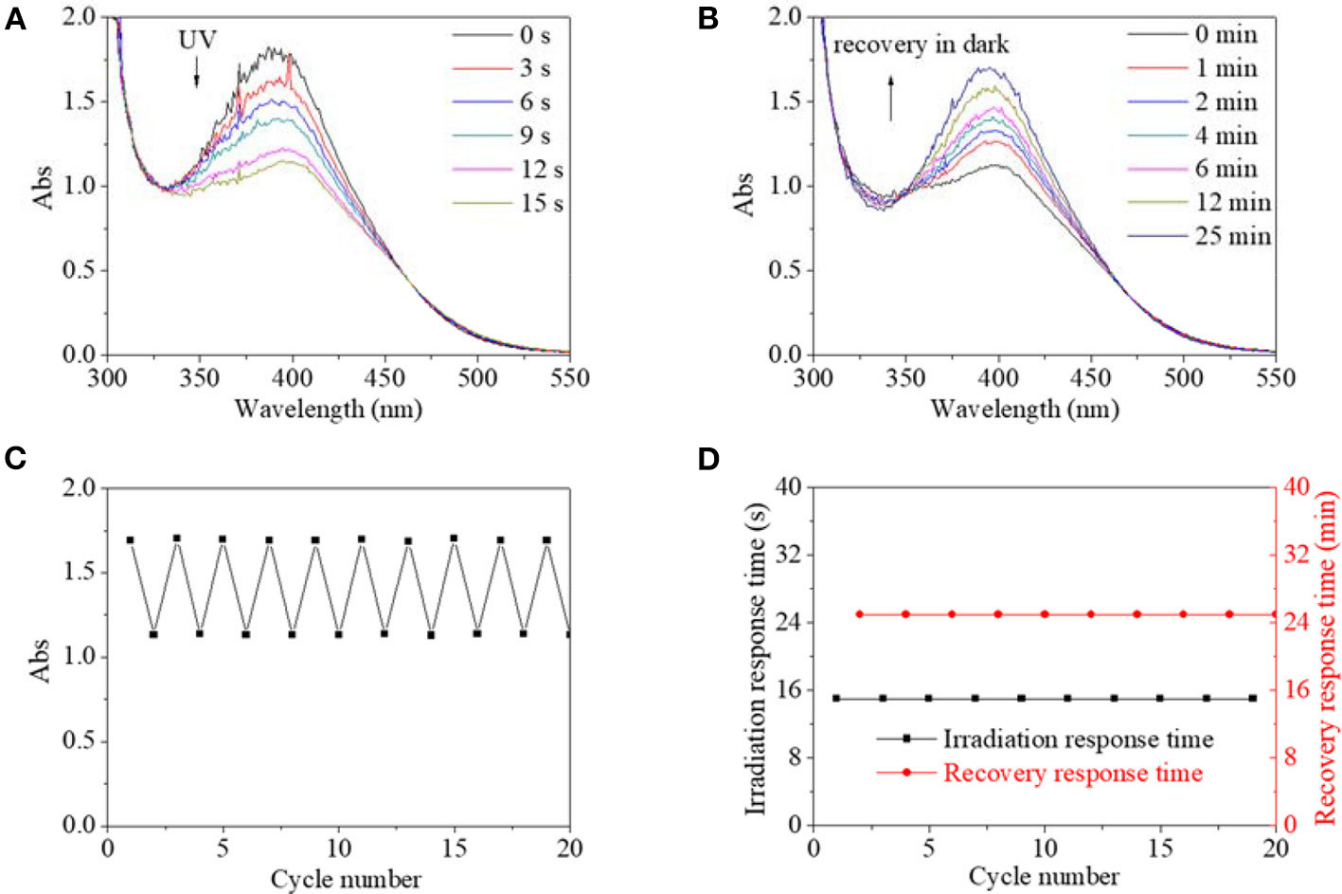
UV spectra of PBLG1 DMF solution as a function of irradiation time **(A)** and recovery time **(B)**. **(C)** Absorbance at 390/400 nm of PBLG1 DMF solution as a function of cycle number. **(D)** Irradiation response time and recovery response time as a function of cycle number. Irradiation was performed under UV light, and the recovery process was performed under darkness and room temperature. Polymer concentration was 17 mg/mL.

Since reversible response and recovery performance were indispensable properties for photo switches, they were investigated in detail. Four excitation sources were firstly used to excite isomerization transformation for PBLGs. UV light as a kind of excitation source could induce isomerization transformation of PBLG1, which is shown in Figure 5. Excited by UV light, absorbance intensity at 390 nm decreased rapidly along with irradiation time until the value stabilized at 61% of its original value with an equilibrium time of 15 s and a simultaneous red-shift of the absorbance peak to 400 nm (Figure 5A). The phenomenon indicated the successful trans-to-cis transformation of PBLG1 according to the above analysis and existing theory for an AZO structure. After irradiation, the decreased absorbances could gradually recover to their original value in the dark within 25 min, which was indicative of the cis-to-trans recovery process (Figure 5B). Moreover, maximum and minimum absorbance at 390/400 nm of PBLG1 solution as well as response time and recovery time were recorded to evaluate the fatigue by repeated on/off irradiation to the same PBLG1 solution. The maximum absorbance at 390 nm was stabilized around 1.7, and the minimum absorbance at 400 nm was stabilized around 1.7 regardless of cycle number (Figure 5C). Furthermore, the response time was stabilized at 15 s and the recovery time at 25 min regardless of cycle number (Figure 5D). These results confirmed not only good fatigue durability but also controllable stability for PBLG1 as a photo switch. Except for UV light, any other excitation source (blue, yellow, and red light) could not induce isomerization transformation. In conclusion, UV light was the only effective excitation source for PBLG1.

**Figure 6:**
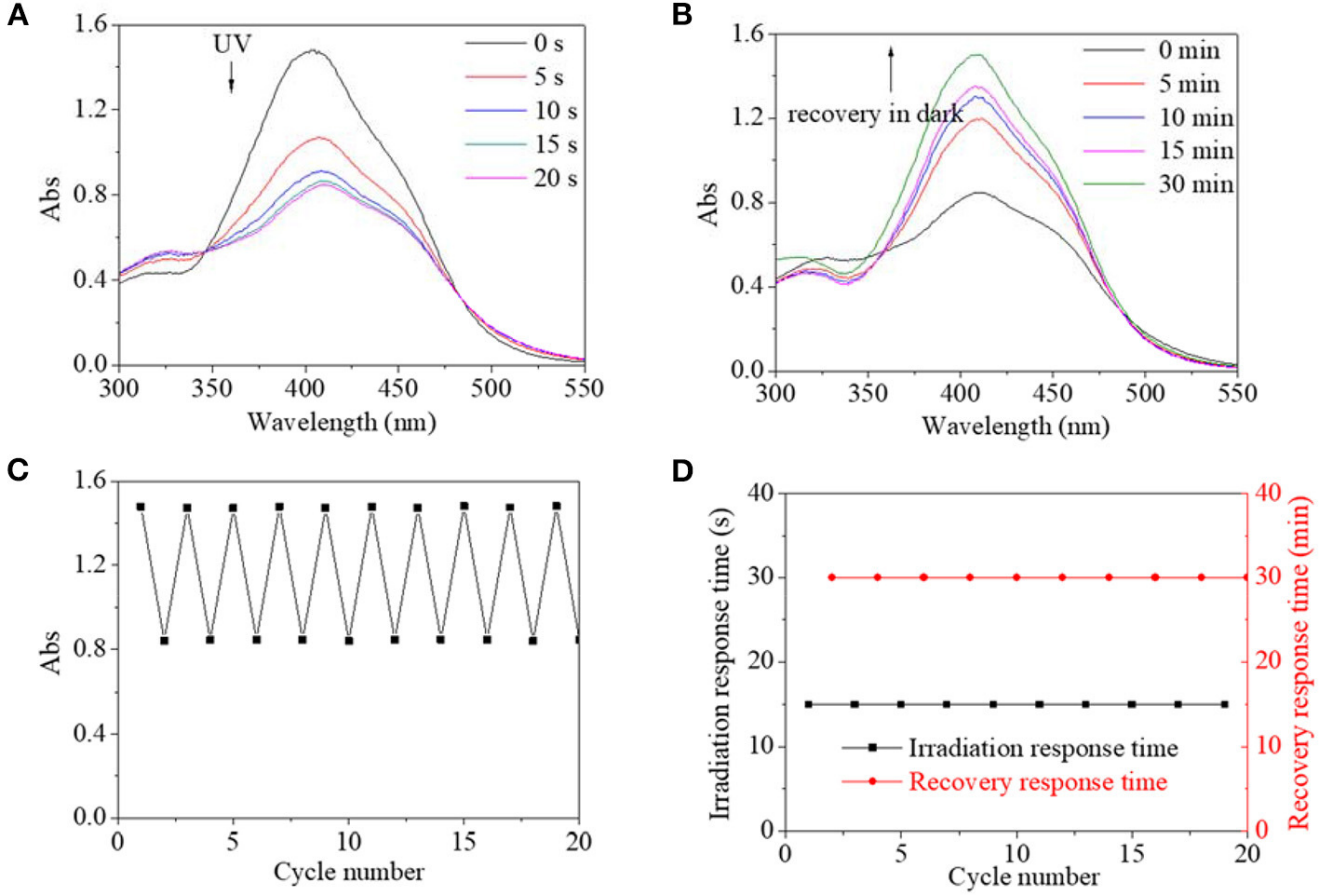
UV spectra of PBLG2 DMF solution as a function of irradiation time **(A)** and recovery time **(B)**. **(C)** Absorbance at 400/410 nm of PBLG2 DMF solution as a function of cycle number. **(D)** Irradiation response time and recovery response time as a function of cycle number. Irradiation was performed under UV light, and recovery process was performed under darkness and room temperature. Polymer concentration was 0.8 mg/mL.

**Figure 7:**
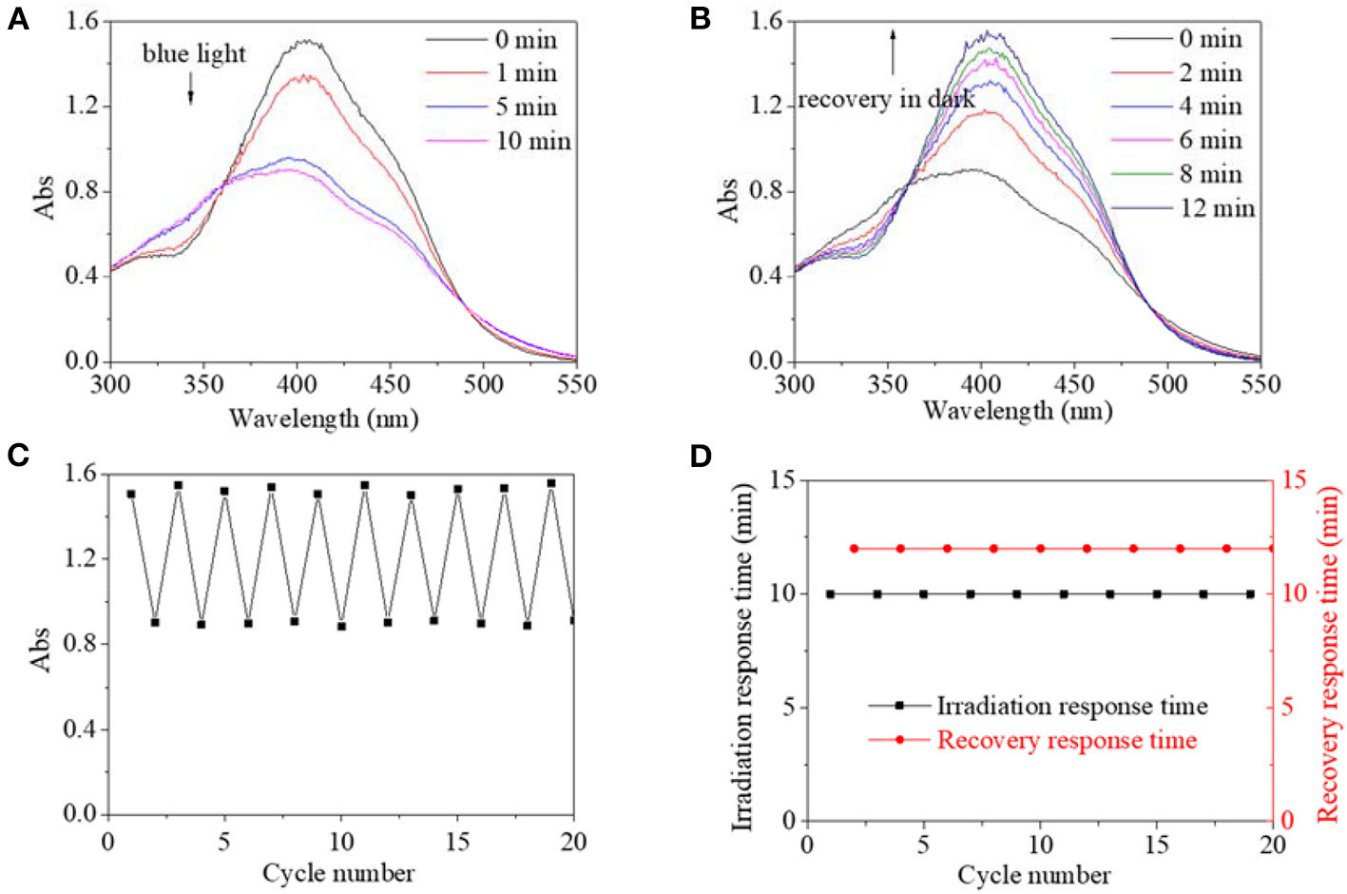
UV spectra of PBLG2 DMF solution as a function of irradiation time **(A)** and recovery time **(B)**. **(C)** Absorbance at 400 nm of PBLG2 DMF solution as a function of cycle number. **(D)** Irradiation response time and recovery response time as a function of cycle number. Irradiation was performed under blue light, and the recovery process was performed under darkness and room temperature. Polymer concentration was 0.8 mg/mL.

Similarly, excited by UV light, the absorbance intensity at 400 nm of PBLG2 solution decreased rapidly from 1.5 to 0.8, about 53% of its original value within an equilibrium time of 15 s; simultaneously, the absorbance peak red-shifted to 410 nm (Figure 6A). After irradiation, the decreased absorbances could gradually recover to their original value in dark within 30 min (Figure 6B). Moreover, by repeated on/off irradiation, the maximum absorbance/the minimum absorbance at 400/410 nm was recorded to stabilize around 1.5/0.8 regardless of cycle number (Figure 6C), and the response time/the recovery time was stabilized at 15 s/30 min regardless of cycle number (Figure 6D). Besides the UV light, blue light could excite trans-to-cis transition for PBLG2, which is shown in Figure 7. Excited by blue light, absorbance intensity at 400 nm of PBLG2 solution decreased gradually from 1.5 to 0.9, about 60% of its original value, within an equilibrium time of 10 min (Figure 7A). After irradiation, the decreased absorbance could gradually recover to their original value in dark within 12 min (Figure 7B). Moreover, the maximum absorbance/ minimum absorbance at 400 nm was stabilized around 1.5/0.9 regardless and the response time/the recovery time was stabilized at 10/15 min regardless of the cycle number (Figures 7C,D). These results confirmed that reversible isomerization transformation for PBLG2 could be effectively excited by both UV and blue light with stable and controllable transition processes and good fatigue durability, which indicated that both UV and blue light were effective excitation sources for PBLG2. Except for UV and blue light, any other excitation source (yellow and red light) could not induce isomerization transformation.

Generally, the effective excitation wavelength of a light source should surround the maximum absorbance wavelength of a UV-vis spectrum for an AZO structure since the UV-vis absorbance peak is directly related to electron transition from the base state to excited state. Simultaneously, combined with the results in the calculation part, PBLG2 exhibited lower excitation energy and higher maximum UV-vis absorbance wavelength. Therefore, PBLG2 could be excited by a light source of a higher wavelength (blue light). Since blue light was a low-energy light source belonging to visible light, PBLG2 exhibited some characteristics of a visible-light-driven photo switch. This characteristic would be beneficial to its potential biomedical application. Thus, PBLG2 as a better photo switch was researched, particularly as it related to considering the solvent and pH effects.

### Solvent Effects on Isomerization for PBLG2

**Figure 8:**
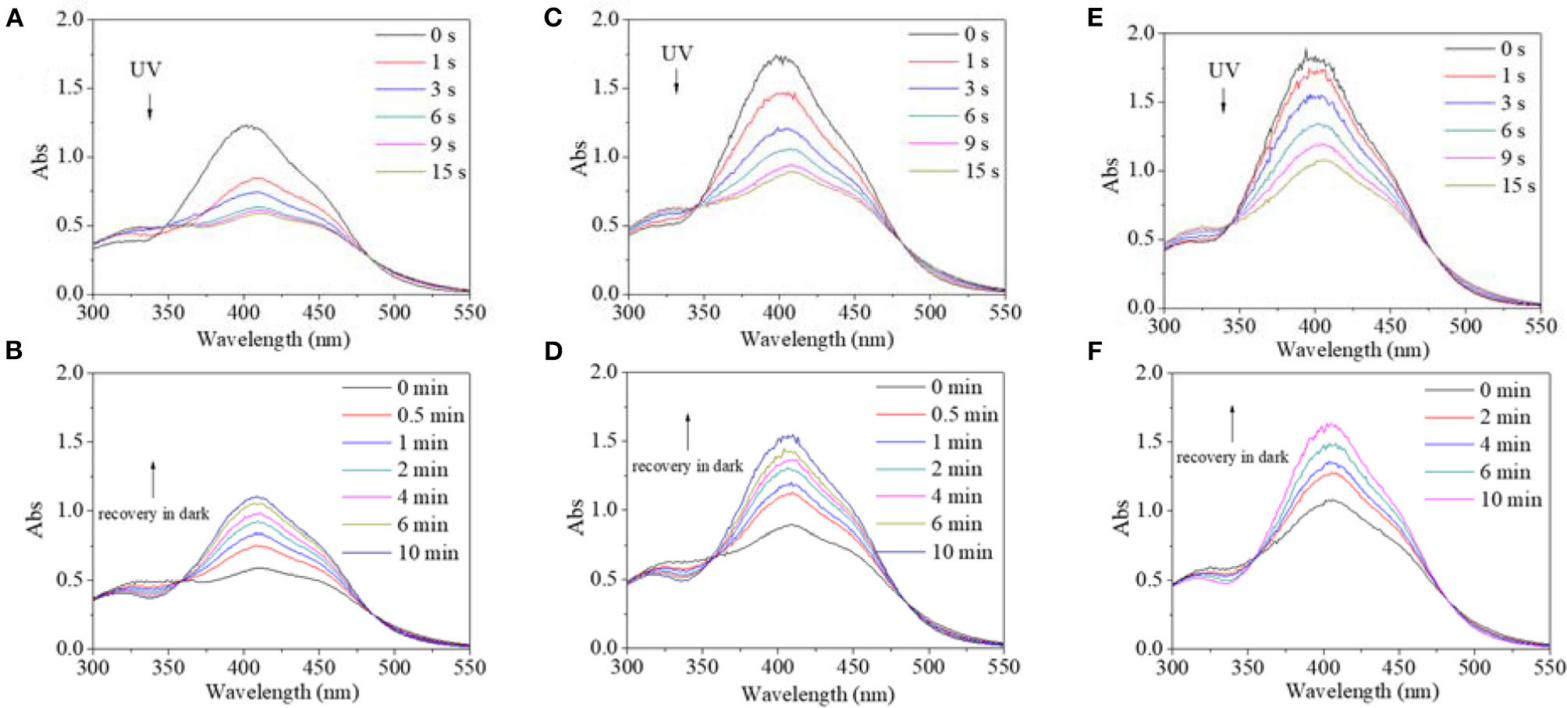
UV spectra of PBLG2 solution with 20% alcohol **(A,B)**, 40% alcohol **(C,D)** and 60% alcohol **(E,F)** as a function of irradiation time (a, c) and recovery time **(B,D)**. Irradiation was performed under UV light, and recovery process was performed under darkness and room temperature. Polymer concentration was 0.65 mg/mL for **(A,B)**, 0.88 mg/mL for **(C,D)**, 0.94 mg/mL for **(E,F)**.

**Figure 9:**
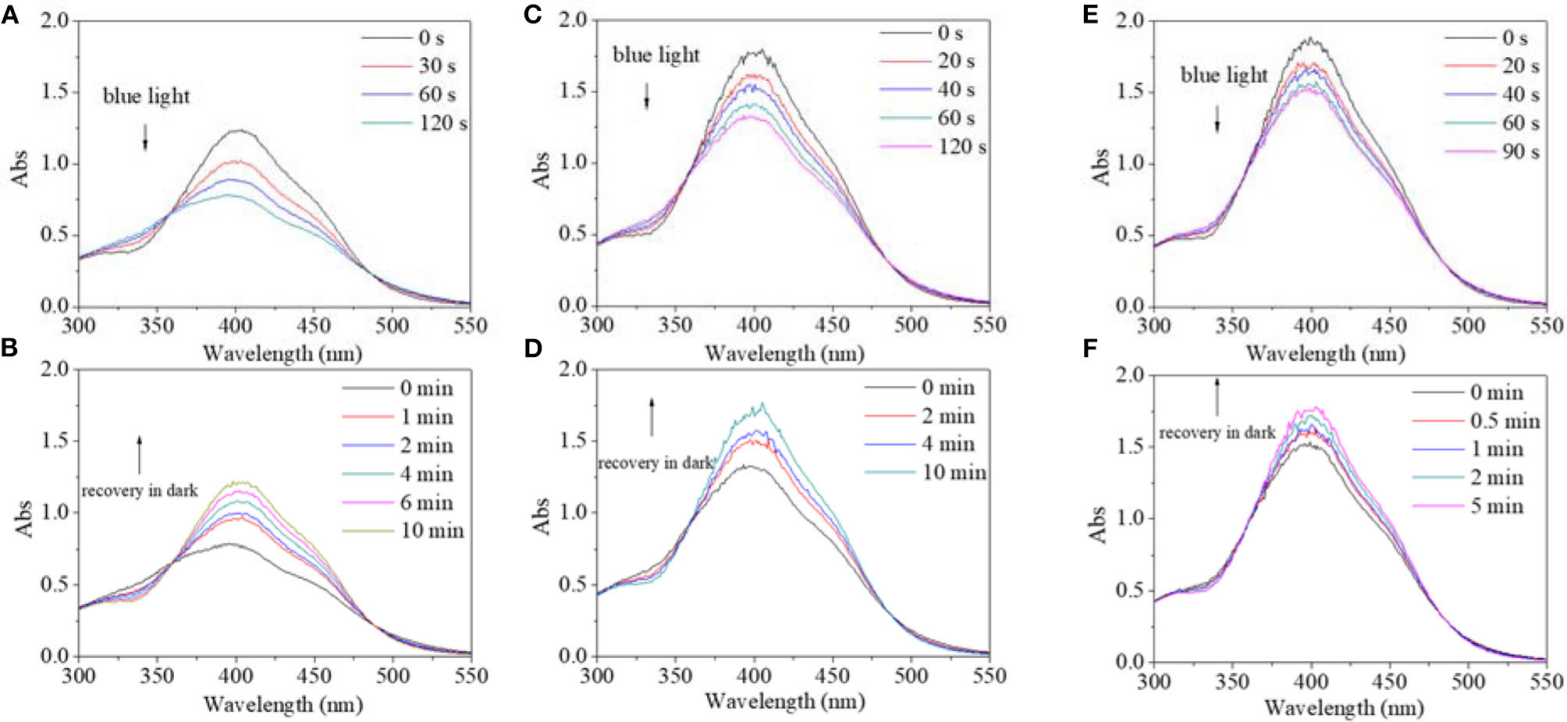
UV spectra of PBLG2 solution with 20% alcohol **(A,B)**, 40% alcohol **(C,D)**, and 60% alcohol **(E,F)** as a function of irradiation time **(A,C)** and recovery time **(B,D)**. Irradiation was performed under blue light, and recovery process was performed under darkness and room temperature. Polymer concentration was 0.65 mg/mL for **(A,B)**, 0.91 mg/mL for **(C,D)**, 0.96 mg/mL for **(E,F)**.

In view of different polarity, alcohol and water were introduced to the solvent system. Alcohol's effect on the trans→cis excitation process was investigated, as seen in Figures 8, 9. Excited by UV light, absorbance intensity decreased rapidly to a constant value (50–55% of the original value) in 15 s in terms of a used solvent containing alcohol, indicating an obvious trans→cis process (Figures 8A,C,E). Likewise, in the dark, decreased values could gradually recover to their original values within 10 min, indicating that there is a cis→trans process (Figures 8B,D,F). Alcohol seemed to have little effect on isomerization transformation initiated by UV light. But the situation was different when blue light was used as excitation source (Figure 9). Upon using blue light, the detectable isomerization process become unclear with the addition of alcohol (Figures 9A,C,E). In more detail, absorbance decreased to 62% of original value within 120 s for the solution with 20/80% alcohol/DMF (Figure 9A), 74% of original value within 120 s for the solution with 40/60% alcohol/DMF (Figure 9C), and 82% of original value within 90 s for the solution with 60/40% alcohol/DMF (Figure 9E). In the dark, the decreased value could gradually recover to its original value, but recovery time shortened with its minimum absorbance after irradiation (Figures 9B,D,F). In more detail, the recovery time was 10 min for the solution with 20/80% alcohol/DMF (Figure 9B), 10 min for the solution with 40/60% alcohol/DMF (Figure 9D), and 5 min for the solution with 60/40% alcohol/DMF (Figure 9E).

**Figure 10:**
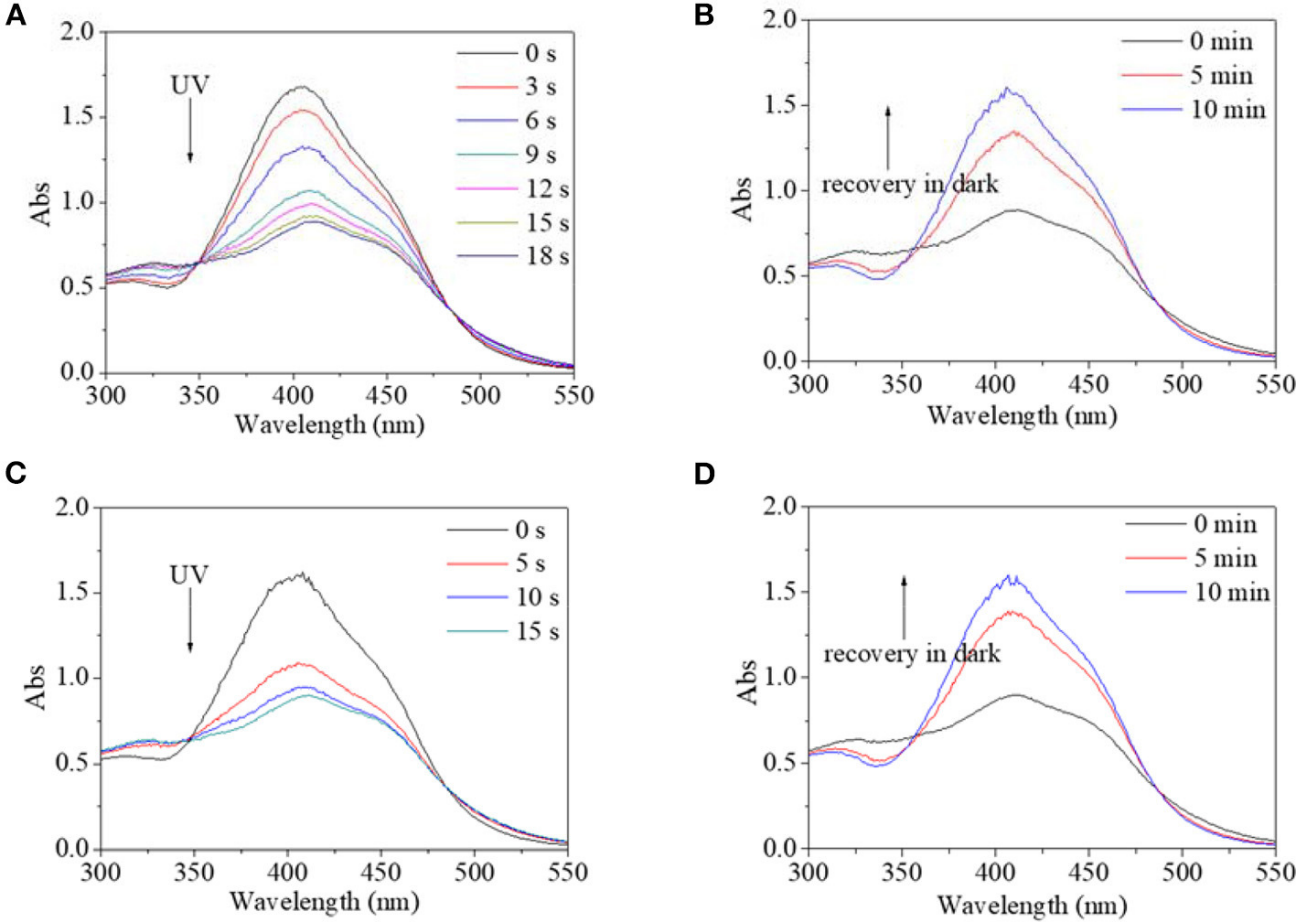
UV spectra of PBLG2 solution with 5% water **(A,B)** and 10% water **(C,D)** as a function of irradiation time **(A,C)** and recovery time **(B,D)**. Irradiation was performed under UV light, and recovery process was performed under darkness and room temperature. Polymer concentration was 0.87 mg/mL for **(A,B)**, 0.83 mg/mL for **(C,D)**.

**Figure 11:**
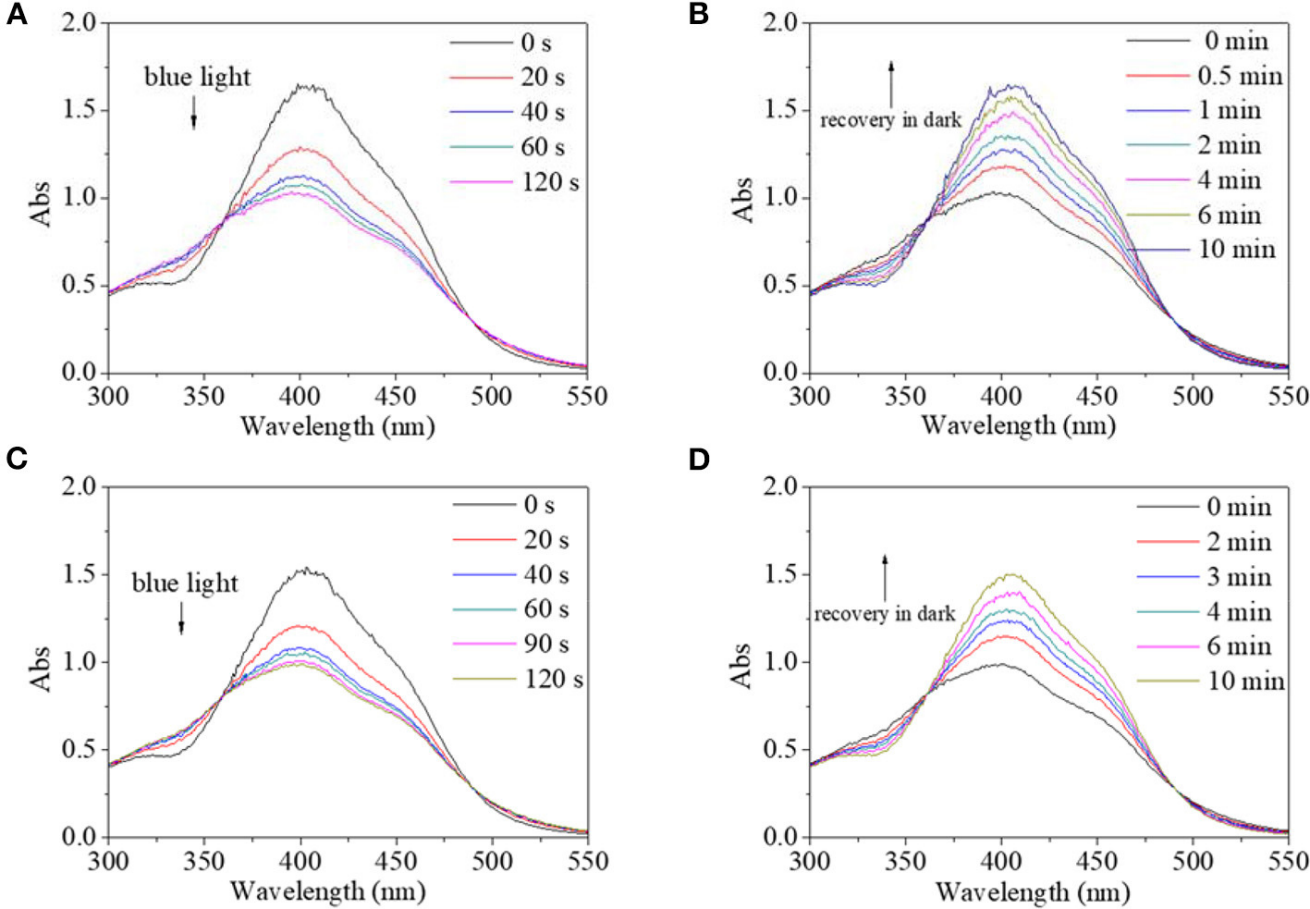
UV spectra of PBLG2 solution with 5% water **(A,B)** and 10% water **(C,D)** as a function of irradiation time **(A,C)** and recovery time **(B,D)**. Irradiation was performed under blue light, and recovery process was performed under darkness and room temperature. Polymer concentration was 0.85 mg/mL.

Besides alcohol, the effect of water on the trans→cis excitation process had similar tendencies (Figures 10, 11). Water content was set to not be higher than 10% because PBLG2 will precipitate if water content is higher than 10%. Excited by UV light, absorbance intensity decreased rapidly to a constant value (50–55% of original value) in 15–18 s regardless of water content (Figures 10A,C). Likewise, in the dark, the decreased value could gradually recover to its original value within 10 min regardless of water content (Figures 10B, D). On account of this similarity, it was inferred that <10% water has little effect on isomerization transformation initiated by UV light. Upon use of blue light, the absorbance at 400 nm decreased to 64% of its original value with the addition of water making it <10% (Figures 11A,B). Also, the deceased absorbance could recover to its original value in the dark (Figures 11C,D). But both response and recovery time in water containing a solvent were shorter than in pure DMF (Figure 11).

In summary, the addition of alcohol and water as cosolvents had little effect on isomerization transformation initiated by UV light except for a shortened recovery time. Furthermore, either alcohol or water in a solvent would accelerate both the trans→cis and cis→trans processes in a blue-light-excited system.

## Conclusion

BLG-NCA was successfully synthesized with a yield of 70–75%. PBLGs were successfully synthesized by use of the NCA method (using m-AZO and m-DAZO as initiators, respectively). DP was calculated to be 14 for PBLG1 initiated by m-AZO and 22 for PBLG2 initiated by m-DAZO. PBLGs exhibited amorphous characteristics with an obvious T_g_ transition, which were 14°C for PBLG1 and 21°C for PBLG2. In calculated UV-vis spectra, PBLG2 had a higher wavelength and larger intensity absorbance peak than PBLG1. Also, the energy gap between HOMO and LUMO for PBLG2 was lower than that for PBLG1, as calculated by DFT. In the experimental system, PBLG1 had a maximum absorbance peak at 390 nm, and PBLG2 had a maximum absorbance peak at 400 nm, red-shifting 40–50 nm from theoretical value. Furthermore, the absorbance intensity of PBLGs had a good linear relationship to their concentration, but the absorbance intensity of PBLG2 was several times that of PBLG1 in the same concentration. Trans→cis transition of PBLG1 could be excited rapidly by UV light. In a dark environment, the cis form could recover to its original trans isomer. Also, good fatigue durability for PBLG1 as a photo switch was confirmed by our results. Other light sources, except for UV light, could not induce detectable isomerization for PBLG1. Upon use of UV light, PBLG2 exhibited similar isomerization characteristic to PBLG1. Blue light, however, was also verified to be another effective excitation source for PBLG2. When it came to solvent effects, the addition of alcohol and water as cosolvents had little effect on the trans→cis transition in UV-light-excited systems, but it shortened the recovery time of cis→trans processes in dark environments. In contrast, the detectable isomerization process becomes unclear with the addition of alcohol in the blue-light-excited system. Furthermore, either alcohol or water in solvents would accelerate both the trans→cis and cis→trans processes in a blue-light-excited system.

## Data Availability Statement

The original contributions presented in the study are included in the article/supplementary material, further inquiries can be directed to the corresponding author/s.

## Author Contributions

PC wrote the manuscript. JK synthesized polymer. XW investigated PBLG1s. WM characterized PBLG2. XY characterized PBLG1. YQ researched the photo responsive properties. XH gave the idea and instructed the whole research. All authors contributed to the article and approved the submitted version.

## Conflict of Interest

The authors declare that the research was conducted in the absence of any commercial or financial relationships that could be construed as a potential conflict of interest.
